# Survival Outcomes in Premenopausal Patients With Invasive Lobular Carcinoma

**DOI:** 10.1001/jamanetworkopen.2023.42270

**Published:** 2023-11-08

**Authors:** Tae In Yoon, Joon Jeong, Seokwon Lee, Jai Min Ryu, Young Joo Lee, Jee Yeon Lee, Ki-Tae Hwang, Hakyoung Kim, Seonok Kim, Sae Byul Lee, Beom Seok Ko, Jong Won Lee, Byung Ho Son, Otto Metzger, Hee Jeong Kim

**Affiliations:** 1 Division of Breast Surgery, Department of Surgery, Dongnam Institute of Radiological & Medical Sciences, Busan, Korea; 2 Department of Surgery, Gangnam Severance Hospital, Yonsei University, Seoul, Korea; 3 Department of Surgery, Biomedical Research Institute, Pusan National University Hospital, School of Medicine, Pusan National University, Busan, Korea; 4 Division of Breast Surgery, Department of Surgery, Samsung Medical Center, Sungkyunkwan University School of Medicine, Seoul, Korea; 5 Division of Breast Surgery, Department of Surgery, Seoul St Mary’s Hospital, College of Medicine, The Catholic University of Korea, Seoul, Korea; 6 Department of Surgery, School of Medicine, Kyungpook National University, Kyungpook National University Chilgok Hospital, Daegu, Korea; 7 Department of Surgery, Seoul National University College of Medicine, Seoul Metropolitan Government–Seoul National University, Boramae Medical Center, Seoul, Korea; 8 Department of Surgery, Dongguk University College of Medicine, Dongguk University Ilsan Hospital, Goyang, Korea; 9 Department of Clinical Epidemiology and Biostatics, University of Ulsan College of Medicine, Asan Medical Center, Seoul, Korea; 10 Division of Breast, Department of Surgery, University of Ulsan College of Medicine, Asan Medical Center, Seoul, South Korea; 11 Department of Medical Oncology, Dana-Farber Cancer Institute, Harvard Medical School, Boston, Massachusetts

## Abstract

**Importance:**

The disparate prognostic implications between invasive ductal carcinoma (IDC) and invasive lobular carcinoma (ILC) have been demonstrated. However, information on premenopausal patients remains insufficient.

**Objective:**

To examine long-term survival outcomes of ILC and IDC in premenopausal patients using national databases.

**Design, Setting, and Participants:**

This cohort study used the Surveillance, Epidemiology, and End Results (SEER), Korean Breast Cancer Registry (KBCR), and Asan Medical Center Research (AMCR) databases to identify premenopausal patients with stage I to III ILC or IDC between January 1, 1990, and December 31, 2015. The median follow-up time was 90 (IQR, 40-151) months in the SEER database, 94 (IQR, 65-131) months in the KBCR database, and 120 (IQR, 86-164) months in the AMCR database. Data were analyzed from January 1 to May 31, 2023.

**Main Outcomes and Measures:**

The primary outcome was breast cancer–specific survival (BCSS), which was analyzed according to histological type, and the annual hazard rate was evaluated. Survival rates were analyzed using a log-rank test and a Cox proportional hazards regression model with time-varying coefficients. Multivariable analysis was performed by adjusting for tumor characteristics and treatment factors.

**Results:**

A total of 225 938 women diagnosed with IDC or ILC and younger than 50 years were identified. Mean (SD) age at diagnosis was 42.7 (5.3) years in the SEER database, 41.8 (5.5) years in the KBCR database, and 41.8 (5.5) years in the AMCR database. In terms of race (available for the SEER database only), 12.4% of patients were Black, 76.1% were White, 11.0% were of other race (including American Indian or Alaska Native, Asian, and Native Hawaiian or Other Pacific Islander), and 0.5% were of unknown race). Patients with ILC had better BCSS in the first 10 years after diagnosis than those with IDC (hazard ratios [HRs], 0.73 [95% CI, 0.68-0.78] in the SEER database, 1.20 [95% CI, 0.91-1.58] in the KBCR database, and 0.50 [95% CI, 0.29-0.86] in the AMCR database), although BCSS was worse after year 10 (HRs, 1.80 [95% CI, 1.59-2.02] in the SEER database, 2.79 [95% CI, 1.32-5.88] in the KBCR database, and 2.23 [95% CI, 1.04-4.79] in the AMCR database). Similar trends were observed for hormone receptor–positive tumors (HRs, 1.55 [95% CI, 1.37-1.75] in the SEER database, 2.27 [95% CI, 1.01-5.10] in the KBCR database, and 2.12 [95% CI, 0.98-4.60] in the AMCR database). Considering the annual hazard model of BCSS, IDC events tended to decline steadily after peaking 5 years before diagnosis. However, the annual peak event of BCSS was observed 5 years after diagnosis for ILC, which subsequently remained constant.

**Conclusions and Relevance:**

These findings suggest that premenopausal women with ILC have worse BCSS estimates than those with IDC, which can be attributed to a higher late recurrence rate of ILC than that of IDC. Histological subtypes should be considered when determining the type and duration of endocrine therapy in premenopausal women.

## Key Points

**Question:**

How do survival outcomes of invasive lobular carcinoma (ILC) differ from those of invasive ductal carcinoma (IDC) in premenopausal patients?

**Findings:**

In this cohort study of 225 938 premenopausal patients, the breast cancer–specific survival of among patients with ILC was significantly worse than that of patients with IDC within the first 10 years after diagnosis.

**Meaning:**

These findings suggest that among premenopausal women, patients with ILC have worse long-term survival outcomes than patients with IDC, and histological subtypes should be considered when determining the type and duration of endocrine therapy in premenopausal women.

## Teaser

This cohort study uses data from 2 large national registries and a single-center database to compare long-term survival outcomes in premenopausal patients with invasive lobular carcinoma vs those with invasive ductal carcinoma.

## Introduction

Invasive lobular carcinoma (ILC) is the second most common histological subtype, accounting for 5% to 10% of invasive breast cancer. In population-based series, when combined hormonal replacement therapy was widely used, the incidence of ILC was significantly higher than that of invasive ductal carcinoma (IDC).^1^ Invasive lobular carcinoma tends to be clinically bilateral, multifocal, or multicentric; pathologically, it tends to be estrogen receptor (ER) positive and/or progesterone receptor (PR) positive and *ERBB2* (previously *HER2/neu*) negative, accompanied by the presence of E-cadherin.^2,3,4,5,6^ Unlike IDC, the metastasis of ILC can be found at specific sites, including the gastrointestinal tract, cerebrospinal fluid, peritoneal sites, pelvic organs, and leptomeninges.^7,8^

Several previous studies have compared the prognosis of ILC with that of IDC. The clinical outcomes of ILC were found to be diverse and better,^3,6^ not different^9,10^ or worse^11^ than those of IDC. Considering the long-term outcome, ILC has been suggested to have a poorer prognosis than IDC.^12,13^ These discrepancies regarding prognosis could be attributed to the different observation periods, particularly considering long-term follow-up and late recurrence.^14^

Despite varying treatment responses documented in the literature, ILC and IDC are addressed using the same standard treatment. Given that most patients with ILC present with low-grade, ER-positive tumors, chemotherapy may not provide substantial benefits.^15^ A retrospective analysis to compare the relative effectiveness of letrozole and tamoxifen in patients with IDC or ILC^16^ used data from the Breast International Group 1-98 trial and found that effects of adjuvant letrozole therapy could differ depending on the histological subtype and were more substantial in patients with ILC. These findings highlight the need to identify the molecular mechanisms underlying tamoxifen resistance in luminal ILC. However, studies comparing the effectiveness of hormonal therapy for ILC and IDC in young women with breast cancer are lacking.

The National Cancer Institute’s Surveillance, Epidemiology, and End Results (SEER) database covers approximately 28% of the population with cancer in the US.^17^ The Korean Breast Cancer Registry (KBCR) is a registry prospectively maintained by the Korean Breast Cancer Society. In 2014, the register included more than 50% of patients with newly diagnosed breast cancer in Korea. Unfortunately, with the end of the provision of death data from the Ministry of Health and Welfare in Korea, the breast cancer mortality data were only available until December 31, 2011. Asan Medical Center Research (AMCR), Korea’s largest single-center registry, assessed analyzed data to overcome a relatively short observation period and obtain more detailed data.

In some retrospective studies, the limited number of ILC occurrences has been documented, and data on premenopausal women with ILC are insufficient. Thus, comparing survival among young women with IDC and ILC is crucial. Therefore, using population-level data from 2 large national registries (SEER and KBCR) and a single-center database (AMCR), we conducted a detailed study to establish the survival and time trends in young women with ILC over a prolonged period.

## Methods

This study followed the Strengthening the Reporting of Observational Studies in Epidemiology (STROBE) reporting guideline. The study was approved by the Asan Medical Center Institutional Review Board, which waived the need for informed consent owing to the use of deidentified registry data.

**Figure 1.**
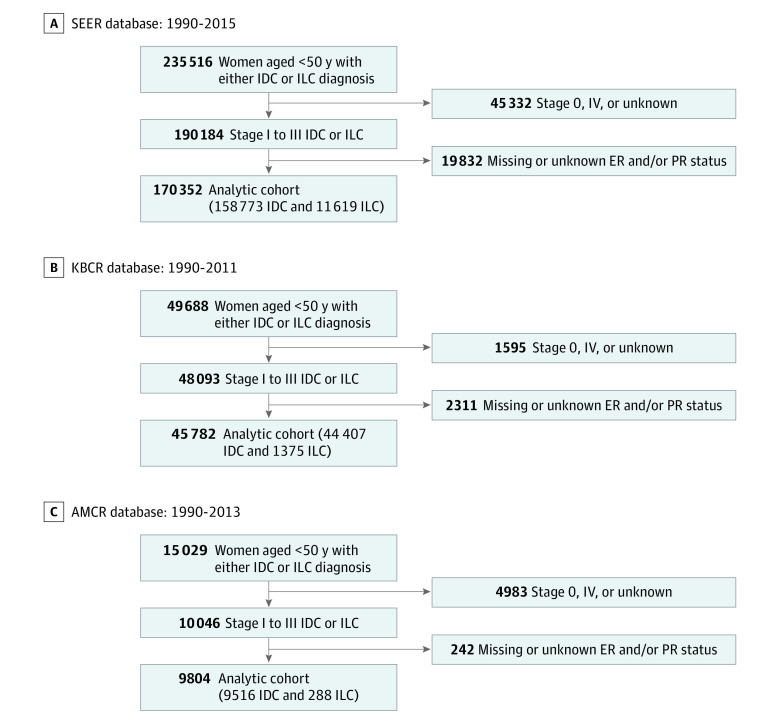
Flow Diagrams for the 3 Study Databases AMCR indicates Asan Medical Center Research; ER, estrogen receptor; IDC, invasive ductal carcinoma; ILC, invasive lobular carcinoma; KBCR, Korean Breast Cancer Registry; PR, progesterone receptor; and SEER, National Cancer Institute’s Surveillance, Epidemiology, and End Results.

Data were obtained from the SEER, KBCR, and AMCR databases. Patients younger than 50 years at diagnosis and with stage I to III pure ILC and IDC were included. Patients for whom information on histology was unavailable or whose hormone receptor status was missing were excluded. Breast cancer–specific survival (BCSS) was analyzed according to histological type and time-dependent BCSS, and the annual hazard rate was evaluated. The primary outcome was breast cancer–related mortality, with other causes of death as competing events. The survival interval was defined as the time from the date of breast cancer diagnosis to either the date of death due to breast cancer or the date of censoring at the last available follow-up. Figure 1 summarizes the study scheme. Data were analyzed from January 1 to May 31, 2023.

### SEER Database

We downloaded data from the SEER 18 registry research database, which contains data from the SEER 13 registry (Atlanta, Georgia; Connecticut; Detroit, Michigan; Hawaii; Iowa; New Mexico; San Francisco–Oakland, California; Seattle–Puget Sound, Washington; Utah; Los Angeles, California; San Jose–Monterey, California; rural Georgia; and the Alaska Native Tumor Registry) and the registries of California, Kentucky, Louisiana, New Jersey, and greater Georgia using SEER*Stat (version 8.3.5). Treatment variables, including chemotherapy and radiotherapy, were collected through additional data consensus. Racial information (Black, White, and other race [includes American Indian or Alaska Native, Asian, and Native Hawaiian or Other Pacific Islander]) were obtained from the SEER registry. The cause of death was determined from the National Death Index data accompanying the SEER file. We identified 235 516 women younger than 50 years with histologically confirmed primary IDC (SEER histological type, code 8500) and ILC (SEER histological type, code 8520) of the breast between January 1, 1990, and December 31, 2015, and who were not diagnosed at autopsy or death. Women with stage 0, IV, or unknown stage cancer (n = 45 332) and unknown ER and/or PR status (n = 19 832) were excluded (Figure 1). The last follow-up was completed on December 31, 2015.

### KBCR Database

Detailed information regarding the web-based system, the KBCR database, has been reported previously.^18^ The KBCR is a database operated by the Korean Breast Cancer Society and is prospectively maintained. To date, breast surgeons from 102 hospitals nationwide have participated in the database. It provides basic information regarding patients, surgical methods, and pathological information, including stages according to the American Joint Committee on Cancer classification system in the *AJCC Cancer Staging Manual*, Eighth Edition; treatment; date of death; and cause of death. The KBCR database does not provide tumor recurrence information, only mortality data. For our study, data regarding survival and cause of death were obtained from the Ministry of Health and Welfare in Korea. Data from 49 688 patients between January 1, 1990, to December 31, 2011, were collected. Women with stage 0, IV, or unknown stage disease (n = 1595) and unknown ER and/or PR status (n = 2311) were excluded (Figure 1). With the end of the provision of national death data, the cause of death could be provided until December 31, 2011. Death due to any cause was provided until December 31, 2014.

### Single-Center Registry

The study retrieved data on patients with breast cancer who received treatment at a single institution from the AMCR, a database system that prospectively collects information on all patients who underwent breast cancer surgery at the Asan Medical Center since 1989. Currently, the database contains information regarding approximately 40 000 patients with breast cancer. It provides information on clinical and pathological features of breast tumors, treatment, breast cancer recurrence, and death details. We collected data from 15 029 patients diagnosed with breast cancer between January 1, 1990, and December 31, 2013, and after exclusion, 9804 were included in the analysis (Figure 1). The final follow-up for the surviving patients was completed on December 31, 2016.

### Statistical Analysis

We used the χ^2^ test to compare differences in characteristic variables between ILC and IDC. The Kaplan-Meier method was used to plot a survival curve. The proportional hazards assumption was confirmed by examining log (−log [survival]) curves and using the Schoenfeld residual test. Nonproportional hazards for histological types were observed in the whole cohort and hormone receptor–positive subcohorts. A Cox proportional hazards regression model with time-varying coefficients based on 10 years was used to model the nonproportional hazards of histological types for BCSS. We performed multivariable analyses by adjusting for tumor characteristics—including age, stage at diagnosis, tumor grade, and hormone receptor status—and additionally adjusting for receipt of adjuvant chemotherapy and radiotherapy. In the SEER registry, we additionally adjusted for race. Statistical significance was set at *P* < .05; all statistical tests were 2-sided. The analysis was conducted using SAS, version 9.4 (SAS Institute Inc) and R, version 3.6.1 (R Project for Statistical Computing).

## Results

Overall, 170 352 patients in the SEER database (158 733 with IDC and 11 619 with ILC), 45 782 in the KBCR database (44 407 with IDC and 1375 with ILC), and 9804 in the AMCR database (9516 with IDC and 288 with ILC) were included in the analysis. The mean (SD) age was 42.7 (5.3) years in the SEER database, 41.8 (5.5) years in the KBCR database, and 41.8 (5.5) years in the AMCR database. The median follow-up time was 90 (IQR, 40-151) months in the SEER database, 94 (IQR, 65-131) months in the KBCR database, and 120 (IQR, 86-164) months in the AMCR database. In terms of race (available for the SEER database only), 12.4% of patients were Black, 76.1% were White, 11.0% were of other race (including American Indian or Alaska Native, Asian, and Native Hawaiian or Other Pacific Islander), and 0.5% were of unknown race. Considering the study population, ILCs accounted for 6.8% of cases in the SEER registry, 3.0% in the KBCR database, and 2.9% in the AMCR database. Table 1 summarizes the baseline characteristics of the cohorts with ILC and IDC. Compared with patients with IDC, those with ILC were significantly older, had lower grades, had more advanced cancer stages, and were more likely to have hormone receptor–positive and *ERBB2*-negative breast cancer. Patients with ILC underwent more mastectomies and received more hormone therapy than those with IDC.

**Table 1.** Baseline Characteristics of the Study Patients

Characteristic	Cancer database by histological cancer subtype^a^								
	SEER			KBCR			AMCR		
	IDC (n = 158 733)	ILC (n = 11 619)	*P* value	IDC (n = 44 407)	ILC (n = 1375)	*P* value	IDC (n = 9516)	ILC (n = 288)	*P* value
Follow-up time, mean (SD), mo	98.1 (73.2)	95.9 (70.2)	.11	65.7 (48.2)	60.5 (44.2)	<.001	123.8 (58.6)	115.8 (53.3)	.01
Age at diagnosis, mean (SD), y	42.6 (5.3)	44.7 (3.9)	<.001	41.7 (5.6)	43.7 (4.2)	<.001	41.8 (5.5)	44.1 (3.9)	<.001
Age, y									
<35	18 086 (11.4)	371 (3.2)	<.001	6566 (14.8)	57 (4.1)	<.001	1407 (14.8)	8 (2.8)	<.001
≥35	140 647 (88.6)	11 248 (96.8)		37 841 (85.2)	1318 (95.9)		8109 (85.2)	280 (97.2)	
Race									
Black	20 028 (12.6)	1061 (9.1)	<.001	NA	NA	NA	NA	NA	NA
White	120 013 (75.6)	9602 (82.6)		NA	NA		NA	NA	
Other^b^	17 922 (11.3)	893 (7.7)		NA	NA		NA	NA	
Unknown	770 (0.5)	63 (0.5)		NA	NA		NA	NA	
Cancer stage									
I	63 457 (40.0)	4241 (36.5)	<.001	16 927 (38.1)	482 (35.1)	<.001	3855 (40.5)	106 (36.8)	.45
II	68 790 (43.3)	4617 (39.7)		20 517 (46.2)	633 (46.0)		4348 (45.7)	140 (48.6)	
III	26 486 (16.7)	2761 (23.8)		6963 (15.7)	260 (18.9)		1313 (13.8)	42 (14.6)	
Tumor grade									
G1 and G2	75 353 (47.5)	7913 (68.1)	<.001	23 865 (53.7)	655 (47.6)	<.001	5624 (59.1)	210 (72.9)	<.001
G3	74 037 (46.6)	1172 (10.1)		14 899 (33.6)	98 (7.1)		3298 (34.7)	14 (4.9)	
Unknown	9343 (5.9)	2534 (21.8)		5643 (12.7)	622 (45.2)		594 (6.2)	64 (22.2)	
Hormone receptor status									
Positive	116 862 (73.6)	11 218 (96.5)	<.001	31 889 (71.8)	1266 (92.1)	<.001	6683 (70.3)	277 (96.2)	<.001
Negative	41 871 (26.4)	401 (3.5)		12 518 (28.2)	109 (7.9)		2833 (29.8)	11 (3.8)	
*ERBB2* status									
Positive	10 981 (6.9)	196 (1.7)	<.001	7573 (17.1)	62 (4.5)	<.001	2101 (22.1)	18 (6.3)	<.001
Negative	39 935 (25.2)	3862 (33.2)		26 159 (58.9)	1010 (73.5)		5900 (62.0)	236 (81.9)	
Unknown	107 817 (67.9)	7561 (65.1)		10 675 (24.0)	303 (22.0)		1515 (15.9)	34 (11.8)	
Surgery									
Breast conserving	NA	NA	NA	21 940 (49.4)	550 (40.0)	<.001	5065 (53.2)	127 (44.1)	.01
Mastectomy	NA	NA		21 675 (48.8)	800 (58.2)		4445 (46.7)	161 (55.9)	
Unknown	NA	NA		792 (1.8)	25 (1.8)		6 (0.1)	0	
Radiotherapy									
Yes	81 054 (51.1)	5473 (47.1)	<.001	24 394 (54.9)	699 (50.8)	<.001	6125 (64.4)	168 (58.3)	.09
No	77 679 (48.9)	6146 (52.9)		13 955 (31.4)	512 (37.2)		3337 (35.1)	119 (41.3)	
Unknown	0	0		6058 (13.6)	164 (11.9)		54 (0.6)	1 (0.3)	
Chemotherapy									
Yes	105 515 (66.5)	6840 (58.9)	<.001	32 846 (74.0)	993 (72.2)	.03	6480 (68.1)	183 (63.5)	.26
No	53 218 (33.5)	4779 (41.1)		7406 (16.7)	265 (19.3)		2972 (31.2)	103 (35.8)	
Unknown	0	0		4155 (9.4)	117 (8.5)		64 (0.7)	2 (0.7)	
Hormone therapy									
Yes	NA	NA	NA	26 643 (60.0)	1063 (77.3)	<.001	6801 (71.5)	277 (96.2)	<.001
No	NA	NA		10 676 (24.0)	129 (9.4)		2613 (27.5)	10 (3.5)	
Unknown	NA	NA		7088 (16.0)	183 (13.3)		102 (1.1)	1 (0.3)	

Abbreviations: AMCR, Asan Medical Center Research; IDC, invasive ductal carcinoma; ILC, invasive lobular carcinoma; KBCR, Korean Breast Cancer Registry; NA, not applicable; SEER, National Cancer Institute’s Surveillance, Epidemiology, and End Results.

^a^ Unless otherwise indicated, data are expressed as No. (%) of patients. Percentages have been rounded and may not total 100.

^b^ Includes American Indian or Alaska Native, Asian, and Native Hawaiian or Other Pacific Islander.

**Figure 2.**
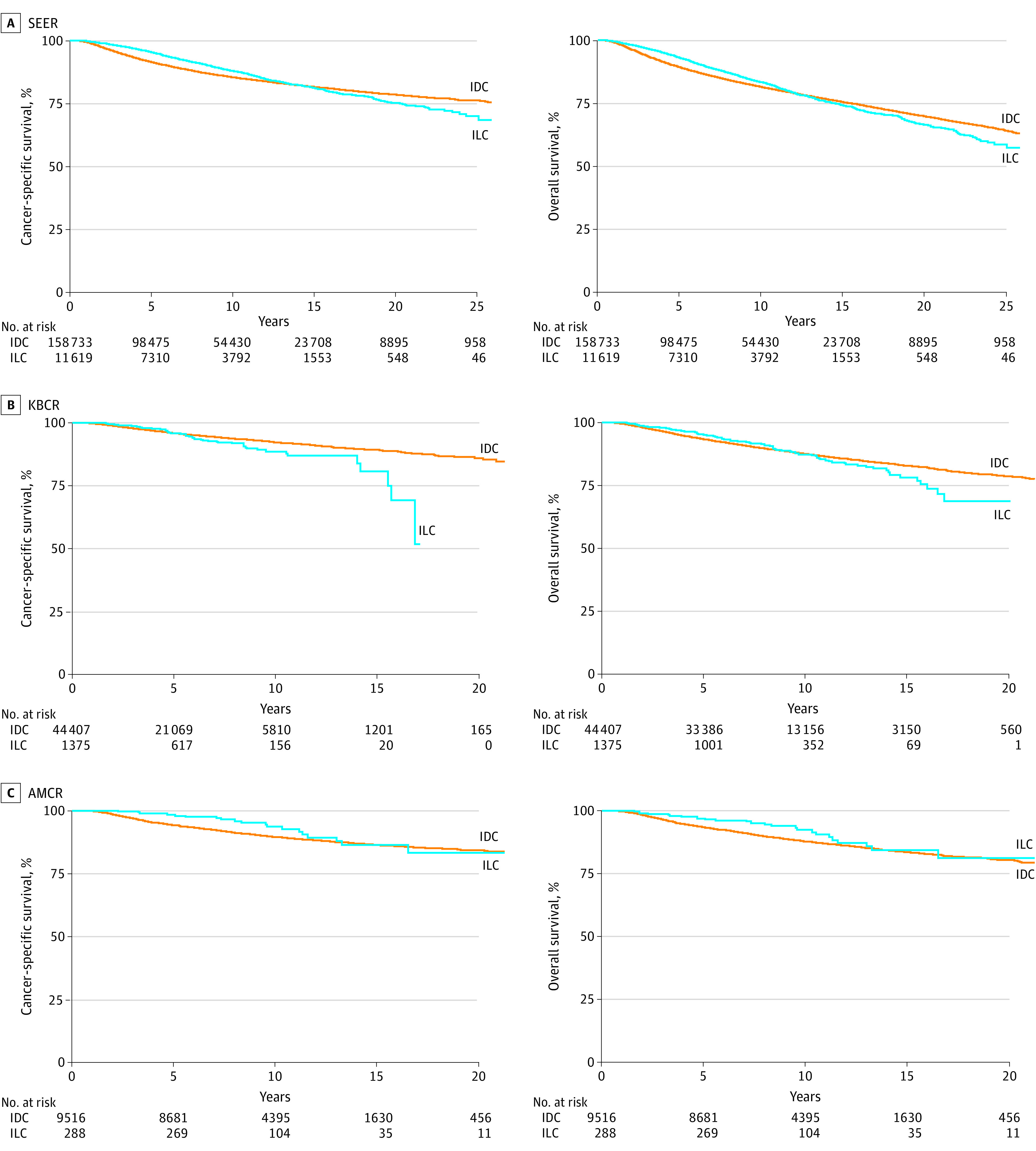
Kaplan-Meier Curves of Breast Cancer–Specific and Overall Survival According to Histological Type AMCR indicates Asan Medical Center Research; IDC, invasive ductal carcinoma; ILC, invasive lobular carcinoma; KBCR, Korean Breast Cancer Registry; and SEER, National Cancer Institute’s Surveillance, Epidemiology, and End Results.

The Kaplan-Meier analysis of BCSS initially favored ILC over IDC, but this trend reversed after a 10-year follow-up (Figure 2). The histological type exerted a statistically significant time-dependent association with BCSS, with ILC decreasing over time in the SEER database (time interaction hazard ratio [HR], 1.93 [95% CI, 1.78-2.10]; *P* < .001). Although other data achieved no statistical significance, a similar pattern was noted.

We performed a similar analysis according to age group (<35 and ≥35 years) using the SEER registry. Patients 35 years and older showed a similar pattern with total group. However, among women younger than 35 years with ILC, we did not observe better survival compared with women with IDC, even during earlier periods (eFigure in Supplement 1).

A Cox proportional hazards regression model was used to estimate change points in BCSS within one interval covering less than 10 years and another covering 10 or more years. Based on the unadjusted model, the risk of BCSS events was 27% lower in the SEER database (HR, 0.73 [95% CI, 0.68-0.78]; *P* < .001) and 50% lower in the AMCR database (HR, 0.50 [95% CI, 0.29-0.86]; *P* = .01) among patients with ILC than those with IDC within the first 10 years after diagnosis (Table 2). After 10 years, patients in the ILC cohort had a higher risk of BCSS events than those in the IDC cohort, with HRs of 1.80 (95% CI, 1.59-2.02; *P* < .001) in the SEER database, 2.79 (95% CI, 1.32-5.88; *P* = .007) in the KBCR database, and 2.23 (95% CI, 1.04-4.79; *P* = .04) in the AMCR database. Similar results were obtained after adjusting for tumor characteristic factors—including age, cancer stage, tumor grade, and hormone receptor status—and after controlling for treatment with chemotherapy and radiotherapy (Table 2). To control the impact of *ERBB2* status and participant therapy, we conducted a separate analysis with single-center data. The result reflecting *ERBB2* status or target therapy also showed that the BCSS of ILC was worse after 10 years (HR, 2.60 [95% CI, 1.16-5.85]; *P* = .02) (eTable in Supplement 1).

**Table 2.** Time-Dependent Outcomes of Breast Cancer–Specific Survival

Histology	No. of patients	No. of events	Survival, mo	Model 1^a^		Model 2^b^		Model 3^c^	
				HR (95% CI)	*P* value	HR (95% CI)	*P* value	HR (95% CI)	*P* value
**SEER**									
Entire cohort									
IDC	158 733	18 889	NA	1 [Reference]	NA	1 [Reference]	NA	1 [Reference]	NA
ILC	11 619	1173	≤120	0.73 (0.68-0.78)	<.001	0.80 (0.74-0.85)	<.001	0.79 (0.74-0.85)	<.001
			>120	1.80 (1.59-2.02)	<.001	1.96 (1.74-2.22)	<.001	1.95 (1.72-2.20)	<.001
HR-positive cohort									
IDC	116 862	11 348	NA	1 [Reference]	NA	1 [Reference]	NA	1 [Reference]	NA
ILC	11 218	1048	≤120	0.87 (0.81-0.94)	<.001	0.84 (0.78-0.91)	<.001	0.84 (0.78-0.90)	<.001
			>120	1.55 (1.37-1.75)	<.001	1.47 (1.30-1.67)	<.001	1.46 (1.29-1.66)	<.001
**KBCR**									
Entire cohort									
IDC	44 407	1961	NA	1 [Reference]	NA	1 [Reference]	NA	1 [Reference]	NA
ILC	1375	71	≤120	1.20 (0.91-1.58)	.19	1.14 (0.86-1.51)	.37	1.15 (0.87-1.52)	.33
			>120	2.79 (1.32-5.88)	.007	2.74 (1.27-5.93)	.01	2.84 (1.31-6.15)	.008
HR-positive cohort									
IDC	31 889	1091	NA	1 [Reference]	NA	1 [Reference]	NA	1 [Reference]	NA
ILC	1266	58	≤120	1.47 (1.08-2.00)	.01	1.18 (0.86-1.61)	.30	1.20 (0.88-1.64)	.26
			>120	2.27 (1.01-5.10)	.05	2.13 (0.95-4.78)	.07	2.19 (0.98-4.91)	.06
**AMCR**									
Entire cohort									
IDC	9516	1011	NA	1 [Reference]	NA	1 [Reference]	NA	1 [Reference]	NA
ILC	288	20	≤120	0.50 (0.29-0.86)	.01	0.63 (0.36-1.08)	.09	0.62 (0.36-1.07)	.08
			>120	2.23 (1.04-4.79)	.04	2.29 (1.05-5.02)	.04	2.24 (1.01-4.97)	.05
HR-positive cohort									
IDC	6683	655	NA	1 [Reference]	NA	1 [Reference]	NA	1 [Reference]	NA
ILC	277	25	≤120	0.79 (0.46-1.37)	.40	0.88 (0.51-1.54)	.66	0.85 (0.49-1.49)	.58
			>120	2.12 (0.98-4.60)	.06	2.09 (0.94-4.65)	.07	1.97 (0.87-4.45)	.11

Abbreviations: AMCR, Asan Medical Center Research; HR, hormone receptor; IDC, invasive ductal carcinoma; ILC, invasive lobular carcinoma; KBCR, Korean Breast Cancer Registry; NA, not applicable; SEER, National Cancer Institute’s Surveillance, Epidemiology, and End Results.

^a^ Unadjusted.

^b^ Adjusted for age (≤35 and >35 years), cancer stage at diagnosis, tumor grade, hormone receptor positivity, and race (only for SEER).

^c^ Adjusted for model 2 covariates and receipt of chemotherapy and radiotherapy.

We analyzed the hormone receptor status separately and found that in patients with hormone receptor–positive cancer, ILC resulted in worse survival than IDC after 10 years of diagnosis, with HRs of 1.55 (95% CI, 1.37-1.75; *P* < .001) in the SEER database, 2.27 (95% CI, 1.01-5.10; *P* = .05) in the KBCR database, and 2.12 (95% CI, 0.98-4.60; *P* = .06) in the AMCR database. The results remained consistent after adjusting for tumor characteristics and treatment factors (Table 2).

**Figure 3.**
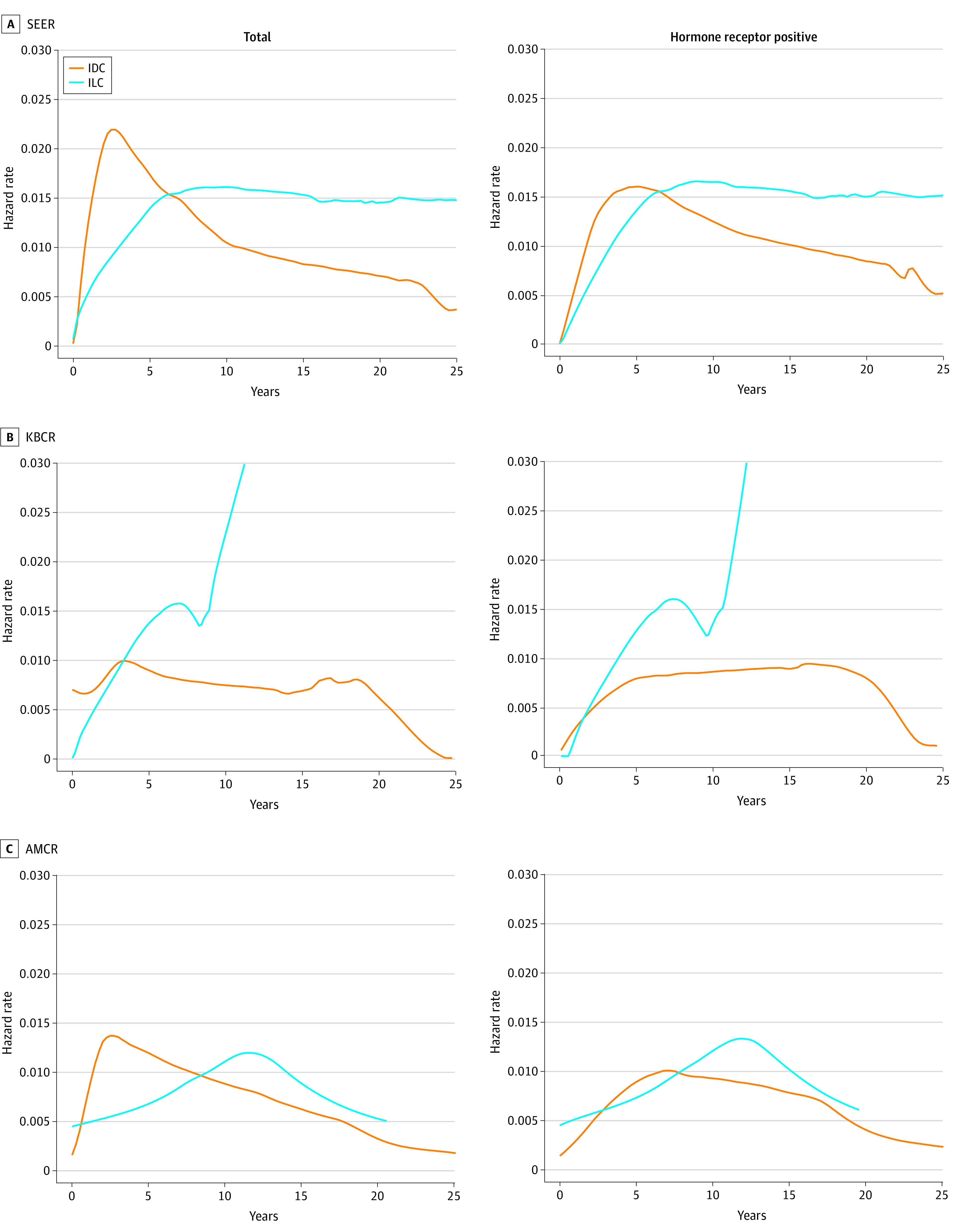
Annual Hazard Rate of Survival Outcome According to Histological Type AMCR indicates Asan Medical Center Research; IDC, invasive ductal carcinoma; ILC, invasive lobular carcinoma; KBCR, Korean Breast Cancer Registry; and SEER, National Cancer Institute’s Surveillance, Epidemiology, and End Results.

In the annual hazard function analysis, IDC peaked at recurrence in the first 5 years after diagnosis, and the hazard rate reduced gradually. Conversely, ILC showed slowly increasing recurrence rates during the initial years, which were maintained for a relatively long time. This observation remained similar when the data were restricted to the cohort with hormone receptor–positive cancer (Figure 3).

## Discussion

This findings of this cohort study suggest that premenopausal women with ILC had a worse time-dependent hazard for BCSS than patients with IDC. Although patients with ILC had better survival during the first 10 years following diagnosis, they experienced poorer BCSS outcomes after 10 years than patients with IDC. Notably, comparable results were obtained in patients with hormone receptor–positive breast cancer.

We found that ILC outcomes tended to be slightly better than those of IDC at the initial interval after diagnosis. However, long-term follow-up revealed a distinct tendency for worse outcomes subsequently. These results were similar to those of previous reports with longer follow-up periods.^19,20,21^ The International Breast Cancer Study Group^20^ conducted 15 prospective adjuvant treatment studies that included 9372 patients classified as having pure IDC (n = 8607) and ILC (n = 767), revealing a substantial initial benefit in the cohort with ILC; however, the cohort with ILC had considerable late losses regarding disease-free and overall survival after 6 and 10 years, respectively. In another large SEER study,^19^ ILC showed early favorable overall survival that worsened after 5 years. Using a large Swedish registry, Chamalidou et al^22^ showed that patients with ILC had improved survival for the first 5 years postoperatively (excess mortality rate ratio, 0.64); however, survival decreased substantially (excess mortality rate ratio, 1.49) 10 to 15 years after diagnosis.

In the present study, specific survival patterns were not associated with hormone receptor status. Hormone receptor–positive breast cancer has been associated with a late recurrence compared with hormone receptor–negative breast cancer; the higher frequency of hormone receptor–positive status in ILC, compared with IDC, may provide a plausible explanation for the observed association of ILC with late recurrence. Enhanced disease-specific survival in patients with ILC might be linked to elevated ER expression, as suggested in previous studies.^6,21^ However, after controlling for and limiting hormone receptor–positive tumors, a similar survival reversal was still observed in later years. In the present study, the ILC survival outcome gradually increased until after 10 years and was maintained. In contrast, IDC showed early recurrence during the first decade, which subsequently stabilized. These results are consistent with those reported by Bouvet et al,^23^ documenting that despite the small number of ILCs included (n = 74), several local recurrences occurred late after the conservation therapy.

In addition to the main analysis, we analyzed patients younger than 35 years using the SEER registry. As in the entire group, the survival rate differed in prognosis over time between IDC and ILC in patients 35 years or older, whereas the survival outcome of ILC was consistently lower than that of IDC in those with breast cancer at younger than 35 years, even in earlier periods (eFigure in Supplement 1). Therefore, various therapeutic approaches should be considered for young patients with ILC.

Young women with breast cancer presented with more aggressive disease than older women with breast cancer. Patients with ILC exhibited lower response rates to chemotherapy than those with IDC. The neoadjuvant studies consistently showed that patients with IDC achieved better chemotherapy responses than those with ILC, with a markedly lower pathological complete response rate.^3,24,25^ After controlling for chemotherapy treatment factors, poor late survival among patients with ILC persisted when compared with patients who had IDC. The National Surgical Adjuvant Breast and Bowel Project B20 trial^26^ used a 21-gene recurrence score to inform decisions on the use of adjuvant chemotherapy in ER-positive breast cancer. Although they did not distinguish between ILC and IDC, identifying patients who would benefit from supplemental chemotherapy compared with endocrine therapy alone may help. Additional studies using multigene assay might be required to confirm the chemotherapy effect of ILC.

The SEER database lacked *ERBB2* status in many cases; therefore, *ERBB2* status was excluded from the analysis. Invasive ductal carcinoma was more likely to be *ERBB2* positive, which could influence outcomes. To compensate for this possibility, *ERBB2* status and target therapy were collected using single institutional data and further analyzed. In an analysis that adjusted *ERBB2* status and targeted therapy, BCSS of ILC still showed worse survival after 10 years. Metzger-Filho et al^27^ demonstrated that ILC was not typically responsive to *ERBB2*-targeted therapy. Additional research on ILC and *ERBB2*-targeted therapy is needed.

Overall, a poor response to systemic treatment, hormone receptor positivity in ILC, and hormonal therapy should be carefully considered to improve outcomes in young women with ILC. Limited information regarding the magnitude of difference in hormone treatment benefits between ILC and IDC, especially in premenopausal women, is available. The 15 International Breast Cancer Study Group clinical trials^28^ included approximately 40% of patients younger than 50 years, and the SEER study by Chen et al^19^ included fewer than 2% of patients with breast cancer younger than 40 years. According to Rakha et al,^21^ endocrine therapy exerts superior benefits in ILC compared with matched IDC; however, women with breast cancer who were younger than 50 years comprised approximately 36% and 24% of the cohorts with IDC and ILC, respectively, and the study lacked information on the type of endocrine treatment.

Our study revealed a consistent risk after 5 years among young women with hormone receptor–positive breast cancer and ILC. The ATLAS (Adjuvant Tamoxifen: Longer Against Shorter) trial^29^ revealed that the effectiveness of tamoxifen persists for approximately 10 years, even after 5 years of administration, and shows a better reduction in relapse and mortality at 10 years of administration. Several endocrine treatment options are available for premenopausal women with breast cancer, including ovarian function suppression plus tamoxifen or aromatase inhibitors. The Suppression of Ovarian Function Trial and Tamoxifen and Exemestane Trial studies^30^ revealed that ovarian suppression combined with aromatase inhibitors could improve overall survival in premenopausal female participants compared with tamoxifen alone or tamoxifen plus ovarian suppression followed up for 8 years. Metzger-Filho et al^16^ demonstrated in a Breast International Group 1-98 study that adjuvant aromatase inhibitors afforded a greater response than tamoxifen in patients diagnosed with ILC vs IDC. Comparing the survival effects of ovarian suppression with aromatase inhibitors between ILC and IDC in premenopausal women with breast cancer would provide clues to guide treatment options in this patient population. Based on this study’s findings, it can be suggested that ILC histology is a determining factor for prolonged treatment or treatment strategies other than tamoxifen therapy. Notably, these results should be cautiously interpreted, as they have been obtained from retrospective analysis.

### Strengths and Limitations

This study’s strength was being the first, to the best of our knowledge, to evaluate the survival of premenopausal patients using 2 large-scale national data sets over prolonged periods. Additionally, to overcome the limitations of national data, a large-scale single-center registry was analyzed to support the results. Accordingly, this will be the cornerstone of future research on the types and duration of hormone therapy for ILC in young female patients.

This study has some limitations. Notably, it was a retrospective study. Although the prevalence of ILC is relatively high in older patients,^1,22^ the incidence of ILC is low, as it affects young patients and involves a small number of patients. Particularly, the prevalence of ILC in patients with hormone receptor–negative breast cancer was low; among them, there were few events, thereby restricting comparisons. Additionally, specific information regarding hormone therapy was lacking. Finally, not only age but also last menstrual period, oophorectomy status, and laboratory tests should be considered to determine a more accurate menopausal status. Owing to limited specific content with national data, the data were categorized by age.

## Conclusions

The findings of this cohort study suggest that the long-term survival of young premenopausal patients with ILC was worse than that of premenopausal patients with IDC. When considering the diverse endocrine therapy options in young female patients, histological subtypes should be considered for selecting endocrine therapy and optimal treatment duration for breast cancer management.

## Notes

Supplement 1. **eFigure.** Kaplan-Meier Curves of Breast Cancer–Specific Survival According to Histological Type and Age **eTable.** Time-Dependent Survival Outcomes of Breast Cancer–Specific Survival in the AMCR Database, Including *ERBB2* Status and Target Therapy

Supplement 2.
Data Sharing Statement
