# Supplementary material for: Survival Outcomes in Premenopausal Patients With Invasive Lobular Carcinoma
Source: JAMA Netw Open. 2023 Nov 8;6(11):e2342270. doi: 10.1001/jamanetworkopen.2023.42270 (PMC10632960; doi:10.1001/jamanetworkopen.2023.42270)
Supplement: Supplement 1. — eFigure. Kaplan-Meier Curves of Breast Cancer–Specific Survival According to Histological Type and Age eTable. Time-Dependent Survival Outcomes of Breast Cancer–Specific Survival in the AMCR Database, Including ERBB2 Status and Target Therapy [file jamanetwopen-e2342270-s001.pdf]

Supplementary Online Content

Yoon TI, Jeong J, Lee S, et al. Survival outcomes in premenopausal patients with invasive lobular carcinoma. *JAMA Netw Open*. 2023;6(11):e2342270. doi:10.1001/jamanetworkopen.2023.42270

eFigure. Kaplan-Meier Curves of Breast Cancer–Specific Survival According to Histological Type and Age

eTable. Time-Dependent Survival Outcomes of Breast Cancer–Specific Survival in the AMCR Database, Including *ERBB2* Status and Target Therapy

This supplementary material has been provided by the authors to give readers additional information about their work.

© 2023 Yoon TI et al. *JAMA Network Open*.

eFigure. Kaplan-Meier Curves of Breast Cancer–Specific Survival According to Histological Type and Age

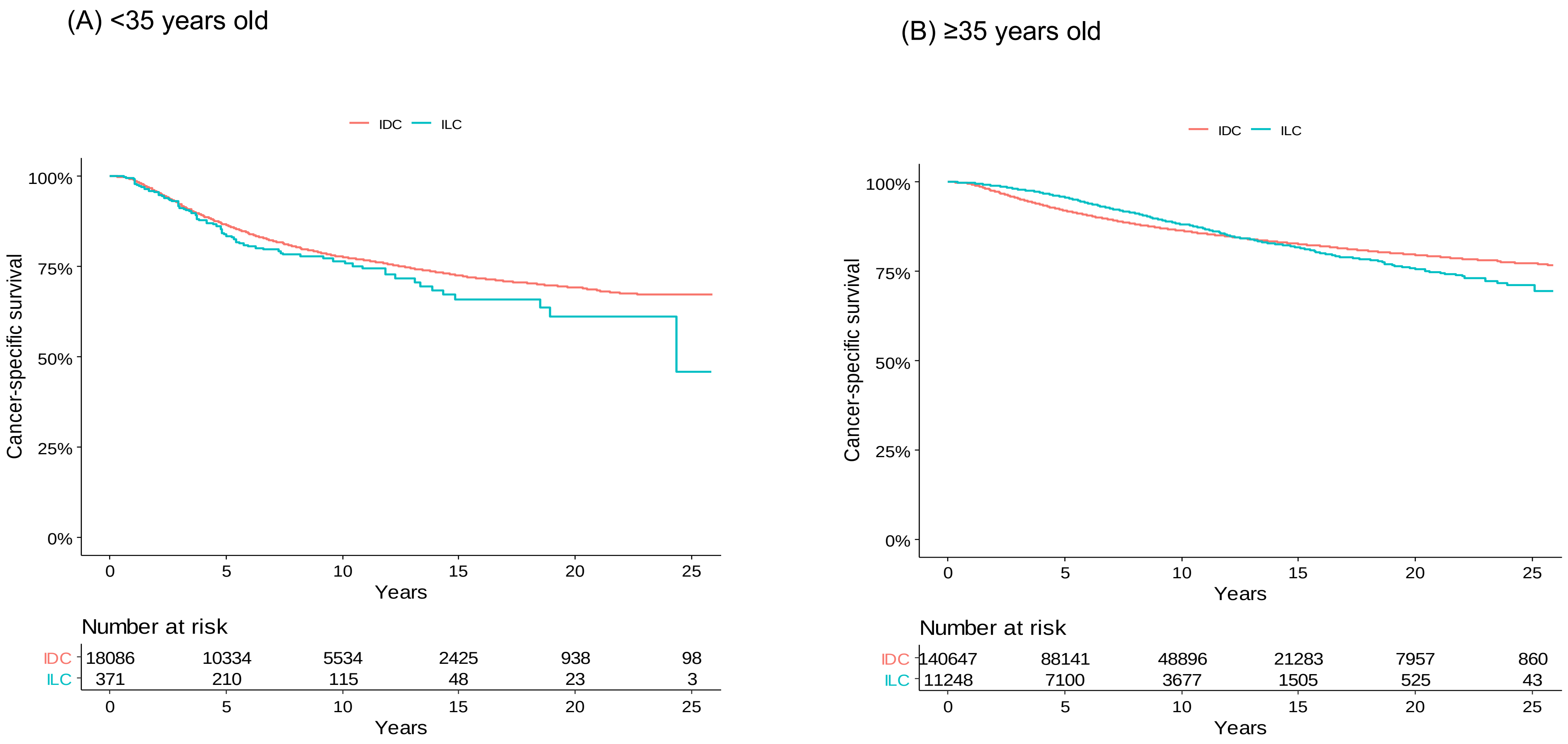

© 2023 Yoon TI et al. *JAMA Network Open*.

eTable. Time-Dependent Survival Outcomes of Breast Cancer–Specific Survival in the AMCR Database, Including *ERBB2* Status and Target Therapy

	Histology	Total	Event	Survival months	HR (95% CI)[1]	p-value	HR (95% CI)[2]	*p*-value	HR (95% CI)[3]	*p*-value
**AMCR**	IDC	9,516	1,011		1		1		1	
	ILC	288	20	≤120	0.497 (0.289-0.855)	.01	0.748 (0.433-1.294)	.30	0.745 (0.430-1.289)	.29
				>120	2.231 (1.038-4.793)	.04	2.570 (1.155-5.179)	.02	2.601 (1.157-5.848)	.02

HR: Hormone receptor, SEER: National Cancer Institute's Surveillance, Epidemiology, and End Results, KBCR, Korean Breast Cancer Registry, AMCR, Asan Medical Center Research; IDC, invasive ductal carcinoma; ILC, invasive lobular carcinoma; CI, confidence interval

[1] Unadjusted

[2] Adjusted for age (<35 and ≥35 years), stage at diagnosis, grade, hormone receptor, and *ERBB2* status.

[3] Adjusted for age (<35 and ≥35 years), stage at diagnosis, grade, hormone receptor, *ERBB2* status, chemotherapy, radiotherapy, and target therapy.

© 2023 Yoon TI et al. *JAMA Network Open*.
