# Supplementary material for: Survival Outcomes in Premenopausal Patients With Invasive Lobular Carcinoma
Source: JAMA Netw Open. 2023 Nov 8;6(11):e2342270. doi: 10.1001/jamanetworkopen.2023.42270 (PMC10632960; doi:10.1001/jamanetworkopen.2023.42270)
Supplement: Supplement 2. — Data Sharing Statement [file jamanetwopen-e2342270-s002.pdf]

Data Sharing Statement

Yoon. Survival Outcomes in Premenopausal Patients With Invasive Lobular Carcinoma. *JAMA Netw Open*. Published November 08, 2023. doi:10.1001/jamanetworkopen.2023.42270

Data
Data available: No
